# The Effect of Zirconia Nanoparticles on Thermal, Mechanical, and Corrosion Behavior of Nanocomposite Epoxy Coatings on Steel Substrates

**DOI:** 10.3390/ma16134813

**Published:** 2023-07-04

**Authors:** Mohammad Asif Alam, Ubair Abdus Samad, Arfat Anis, El-Sayed M. Sherif, Hany S. Abdo, Saeed M. Al-Zahrani

**Affiliations:** 1 Center of Excellence for Research in Engineering Materials (CEREM), King Saud University, Riyadh 11421, Saudi Arabia; esherif@ksu.edu.sa (E.-S.M.S.); habdo@ksu.edu.sa (H.S.A.); 2 SABIC Polymer Research Center (SPRC), Chemical Engineering Department, King Saud University, P.O. Box 800, Riyadh 11421, Saudi Arabia; aarfat@ksu.edu.sa (A.A.); szahrani@ksu.edu.sa (S.M.A.-Z.)

**Keywords:** corrosion protection, coatings, EIS, mechanical properties, thermal behavior

## Abstract

Zirconia (ZrO_2_) nanoparticles (1–3 wt.%) were incorporated into the epoxy matrix using the ultra-sonication mixing method of dispersion to manufacture nanocomposite coatings. An automatic applicator was used to prepare the coating samples on a stainless steel substrate. The influence of ZrO_2_ nanoparticles on the physicochemical characteristics of epoxy coatings was evaluated using energy dispersive X-ray spectroscopy (EDS), field emission scanning electron microscopy (FE-SEM), Fourier-transform infrared spectroscopy (FTIR), thermos-gravimetric analysis (TGA), elastic modulus, and micro-hardness measurement with the nano-indentation technique. The corrosion stability during immersion in 3.5% NaCl solution was monitored using electrochemical impedance spectroscopy (EIS). All ZrO_2_-containing coatings showed better corrosion stability and adhesion than pure epoxy coating. Epoxy coating incorporated with 2% ZrO_2_ exhibited the greatest values of corrosion resistance and adhesion due to the effect of nanoparticle properties and their better de-agglomeration in the epoxy matrix than pure epoxy coating.

## 1. Introduction

With the advent of nanotechnology into the industrial world, epoxy and its composites have become one of the most promising thermoset materials used in the coating formulations industry due to their wide variety of unique properties. Both single and hybrid nano-additives will give the epoxy matrix new and distinct properties that qualify it to produce nanocomposite coatings with powerful properties and can be used for multifarious substrates in many applications under harsh environmental conditions [1]. Epoxy resin filled with inorganic particles, used to fabricate the coatings and composites for various applications, has been a choice for a long time to achieve high performance for different substrates applications. However, in recent decades, nanoparticles (NPs) have shown superior performance, especially in preparing high-performance composites, and received great attention.

Polymeric nanocomposites that are filled or incorporated with nanoparticles exhibit specific features due to the high surface area of the nanoparticles, which subsequently leads to a high contact area between the matrix and the reinforcement. These specific features of the nanocomposite are the main qualifications to use it in a high-performance application. Several previous research proved that metallic or inorganic nanoparticles could be effectively used as a reinforcement for both types of polymeric matrices, thermoplastic and thermosetting [2]. Few studies of epoxy-nanocomposite coating incorporated with -ZrO_2_ nanoparticles have been reported so far to investigate their morphology and mechanical properties. The authors of [3] found out that increasing the content of ZrO_2_ nanoparticles reinforcement in an epoxy matrix proportionally improves its mechanical properties, which can be attributed to the perfect interfacial bonding between the filler and the matrix, and the very high strength of the filler itself. An additional enhancement to the final coating layer is the higher coating toughness, which can be achieved by using amino propyl trimethoxy silane (APS) in treating the zirconia nanoparticles before addition to the epoxy matrix [4]. Moreover, better corrosion resistance performance can be achieved when using a hybrid treatment or combinations of clay and APS in treating the zirconia nanoparticles, which improve the barrier properties and enhance the adhesion through the contact surface between epoxy and zirconia filler [5,6].

The homogeneity of the distribution of the zirconia particles in the epoxy resin is a very important factor in improving the mechanical properties of the structural adhesives matrices with an amine hardener [7]. Rao et al. developed a flame-retardant coating by incorporating zirconia nanoparticles into the polymer matrix. They found that the oxygen index limit increases as the amount of zirconia nanoparticles added increases. They also concluded from the thermal gravimetric analysis (TGA) results that the coating thermal stability at 800 °C is positively affected by increasing the zirconia nanoparticles, therefore qualifying the developed flame retardant coating as having excellent performance and to be applied in many applications [8,9].

In this study, different percentages of zirconia (ZrO_2_) nanoparticles (1, 2, and 3% weight percentages) were used as a reinforcement to the epoxy matrix to be used as a corrosion protection coating layer to the steel substrate. Corrosion performance tests were conducted in 3.5% NaCl solution using three-electrodes electrochemical impedance spectroscopy. The chemical composition and the morphological analysis of the nanofillers were characterized using X-ray spectroscopy (XRD) and field emission scanning electron microscopy (FESEM) with an energy dispersive spectroscopy (EDS) unit attached. The corrosion resistance enhancement of the epoxy with ZrO_2_ reinforcement compared to the neat epoxy without nanofillers has been investigated.

## 2. Material and Methods

Epoxy resin bisphenol-A type (Hexion Chemicals, Duisburg, Germany) was used as the matrix resin, and D-450 (Aradur polyamidoamine adduct) was used as the epoxy curing agent (Huntsman Advanced Materials, Deggendorf, Germany). Wetting and antifoaming agents used were provided in kind by BYK-Chemie GmbH, Wesel, Germany. Solvents such as methyl isobutyl ketone (MIBK) and xylene were utilized to ease nanoparticle mixing in epoxy. ZrO_2_ nanoparticles were procured from Sigma-Aldrich, St. Louis, MO, USA (catalog number: 544,760, <100 nm particle size).

ZrO_2_ nanoparticles (typically 1, 2, and 3 wt.%) were formulated in balanced stoichiometric epoxy and hardener quantities. Firstly, the epoxy resin’s viscosity was reduced with the help of xylene in order to allow the mixing of nanoparticles in resin by utilizing a mechanical high-speed mixer (Sheen S2 disperse master, London, UK). With the help of the ultra-sonication technique, ZrO_2_ NPs were initially dispersed in acetone with the help of silane to achieve good dispersion of NPs in epoxy resin. Sonication was performed for 30 min to fully disperse the NPs in acetone. After finishing sonication, the solution was mixed with the resin under continuous stirring (500 rpm). After adding the solution, the mixing speed was increased to 4000 rpm to achieve uniform nanoparticle dispersion and removal of excess solvent from the system, mixing was performed for 45 min at this high speed. After the mixing was complete, the formulated epoxy resin was left for stabilization for 20 to 30 min. A stoichiometric balanced amount of hardener was then added to the resin and samples were coated for characterization. The complete formulating ingredients along with the quantities used are provided in detail in Table 1.

The morphology of the prepared coatings was analyzed with a field emission scanning electron microscope (FE-SEM), model JSM-7400F (JEOL, Akishima, Japan), along with energy dispersive X-ray spectroscopy (EDS). The Bruker D8 Discover (Billerica, MA, USA) with Cu Kα radiation operated at 40 kV and 40 mA was used to verify the presence of nanoparticles in the prepared coatings. In order to check the effect of nanoparticle addition on thermal properties and decomposition temperatures, thermogravimetric analysis (TGA) was performed using a Q600 by TA instruments (New Castle, DE, USA). The coatings samples were heated up to 600 °C under a N_2_ environment at a heating rate of 10 °C/min in a standard aluminum pan using a ramping rate process.

The pendulum test (model 707/K, Sheen Instruments, Cambridge, UK), which follows the ASTM D-4366 [10], was utilized to measure the coatings’ surface hardness, where the number of oscillations defines the surface hardness, i.e., the higher the number of oscillations is, the higher the hardness. A scratch tester (model 705) was employed to check the scratch resistance of the coatings under the ASTM D-7027 [11] procedure. Furthermore, the resistance to impact for the coatings was estimated using a BYK-Gardener (model IG-1120) following the ASTM D-2794 [12], with a standard falling weight of 8 lb. The results provided for these tests are the average reading from 3 test specimens. Hardness and elastic modulus for all coating formulae were evaluated using a Berkovich-type indenter (Nanotest platform-3, Micromaterials, Wrexham, UK). To determine the coating properties, indentation testing was performed with a controlled load method to determine the coating properties, and each prepared coating was subjected to 250 mN of maximum load. All the tests were performed at a loading rate of 10 mNs-1 followed by a holding period of 60 s, and then unloaded at the same rate until the load was completely removed. To obtain result uniformity, at least 10 indentations were performed, although the final results obtained were averaged.

The EIS data were measured in 3.5% NaCl solutions after various immersion time periods (1 h to 30 days). An Autolab potentiostat (Metrohm, Amsterdam, The Netherlands) was employed at the open circuit potential and between 100,000 to 0.1 Hz as a frequency range and a ±5 mV amplitude sinusoidal wave perturbation.

## 3. Results and Discussion

### 3.1. FE-SEM and EDS Investigations

**Figure 1:**
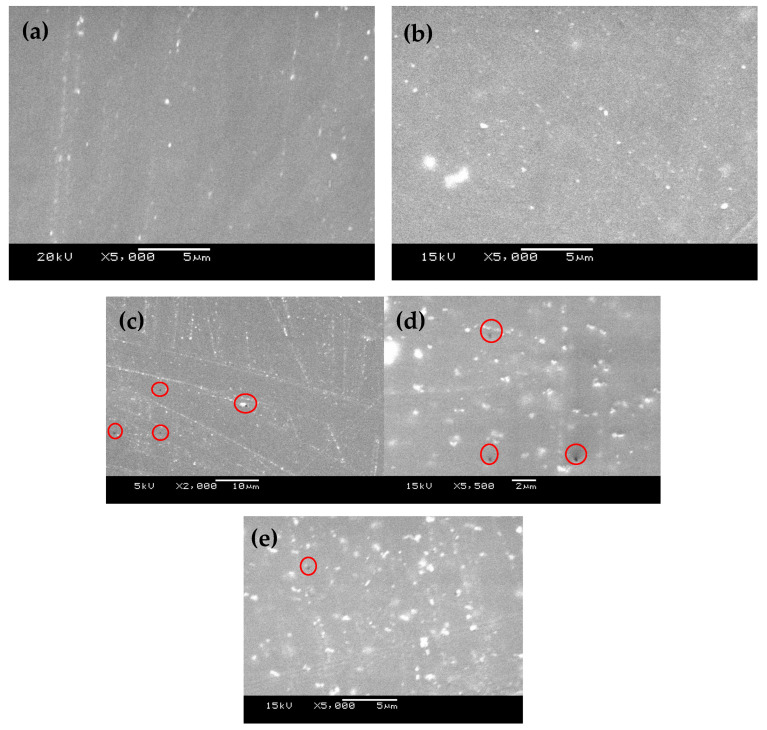
Scanning electron microscope images for prepared coatings (**a**) EZr-1, (**b**) EZr-2, and (**c**–**e**) EZr-3.

**Figure 2:**
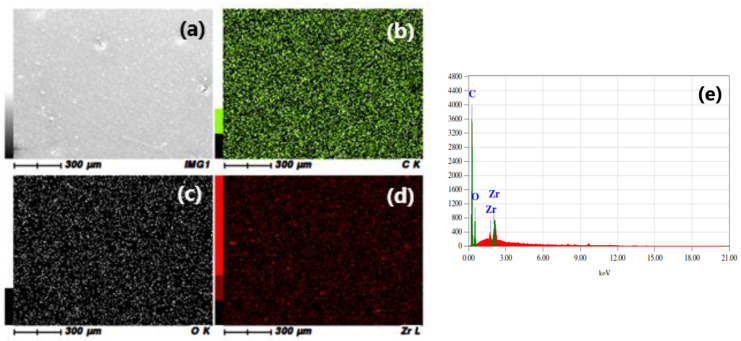
EDS mapping for the epoxy coating containing 2% ZrO_2_ nanoparticles (EZr-2). (**a**) The original SEM image, (**b**) Carbon distribution, (**c**) Oxygen distribution, (**d**) Zirconia distribution, (**e**) EDS pattern.

**Figure 3:**
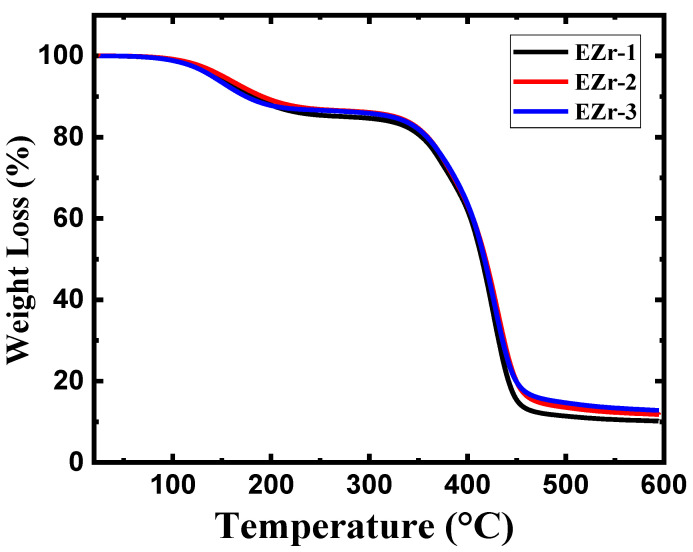
TGA curves of prepared epoxy coatings with different percentages of ZrO_2_.

Figure 1 shows the FE-SEM images taken on the surface of metal coated with epoxy coatings having different percentages of zirconium nanoparticles; here, the images represent (a) EZr-1, (b) EZr-2, and (c–e) EZr-3, respectively. The images depict that the surface is homogenous for all the coating samples with no irregularities. With the increase in the Zr percentage in the coating from 1 wt.% (Figure 1a) to 3 wt.% (Figure 1c), the appearance of the surface is changed. The images show more bright spots with a high percentage of ZrO_2_, due to the addition of Zr nanoparticles in the epoxy coating. It also indicates a change in the morphology of the coatings with the presence of Zr nanoparticles in the epoxy coating. Adding 1 and 2 wt.% Zr nanoparticles resulted in a smooth surface without any voids, whereas pinholes on the coating surface were observed in the presence of 3 wt.% Zr.

EDS analysis was performed to check nanoparticle distribution in the prepared coating samples. Figure 2 shows the EDS mapping results for the EZr-2 coating sample, including (a) the analyzed surface area and (b) the distribution of C, (c) O, and (d) Zr elements; the percentage of C, O, and Zr are also depicted in Figure 2e. It is clear from the mapping images that ZrO_2_ nanoparticles are well distributed across the coating. The formation of aggregates at a very small scale, which is very common with the nanoparticles used, is also visible. The obtained percentages for all coatings are shown in Table 2. Overall, the distribution of nanoparticles is good in our prepared coating samples.

### 3.2. Thermogravimetric Analysis (TGA)

Thermogravimetric analysis for the prepared coatings was performed under a nitrogen gas atmosphere. The purpose of performing analysis under inert gas was to prevent the samples from undergoing thermal oxidation because of the oxygen present in the air. The TGA analysis curves for epoxy coatings modified with the addition of zirconium nanoparticles on thermal properties are shown in Figure 3. The results extracted from these curves are shown in Table 3. A two-step thermal degradation profile is observed in all the prepared samples. The initial stage of degradation starts above 100 °C, while the second stage of degradation started above 300 °C and continued up to 500 °C, where the major changes in chain decomposition happen [13,14]. Table 3 summarizes the temperature extracted from the TGA curves at 15, 25, 50, and 75% weight loss. At the initial first stage of decomposition, the short polymer chains, unreacted residues of both the reactants, and volatiles trapped in the epoxy complex cross-linked structure are degraded. The weight loss at this initial first phase of degradation is approximately 15% for all the prepared coatings.

At stage-2 degradation, the largest decomposition profile in all the prepared coatings involves the decomposition of the main epoxy chain that starts from 300 °C. The weight loss percentages recorded at the end of the experiment were as follows: EZr-1 (90%), EZr-2 (88%), and EZr-3 (87%). Table 3 describes the temperatures at 15, 25, 50, and 75% weight loss. It can be seen in Table 3 that the coating with 1% ZrO_2_ recorded approximately 279 °C at the initial stage of decomposition (15% weight loss), which significantly improved to about 326 °C for both the coatings with 2% and 3% addition. Another significant difference was observed at 75% weight loss, where the temperature values significantly increased to 442 °C with the addition of 2% ZrO_2_ nanoparticles. The TGA data thus confirm that ZrO_2_ nanoparticles improve the thermal properties, and this effect increases with the increase in the nanoparticle percentage.

The increase in the percentage of ZrO_2_ from 1 to 2 wt.% had a pronounced effect on the weight loss temperatures. Upon further increase in the percentage to 3 wt.%, the results were approximately similar to that obtained with a 2 wt.% addition. This increase in degradation temperature is an indication of strong filler/matrix interaction [15]. The ZrO_2_ acts as a thermally insulating material [16], and highly dispersed nanoparticles in the matrix play a vital role in shaping the material properties [17]. The addition of a 2% enhancement in thermal properties is because of the high amount of dispersion achieved, which is confirmed by the SEM images, while the formation of clusters with the addition of 3%, as evidenced by FE-SEM, might be the reason for its underperformance in terms of thermal resistance.

### 3.3. Mechanical Properties and Nanoindentation

The characterization of the developed coating’s mechanical properties with the addition of ZrO_2_ NPs in various percentages was performed using a scratch tester, pendulum hardness, impact strength analyzer, and nanoindentation. The dry film thickness (DFT) for the coatings before the test was recorded using Sheen MiniTest 3100 after complete curing.

With the help of the pendulum tester (Koenig), the surface hardness of the prepared coatings on glass was determined; it was observed that irrespective of added percentage, the hardness of all the prepared samples was the same. The impact strength for all the coatings was the same with different percentage addition of ZrO_2_. The values obtained for all the tests are provided in Table 4. Additionally, the scratch resistance measurement on the coatings with the increasing percentage of ZrO_2_ nanoparticles on steel substrates increased scratch resistance. The addition of ZrO_2_ nanoparticles influences the scratch resistance in the matrix resin. These modifications were performed on the best-performing coatings from our previous work [18]. Based on those results, it is observed that the addition of ZrO_2_ significantly influences the mechanical properties of coatings.

**Figure 4:**
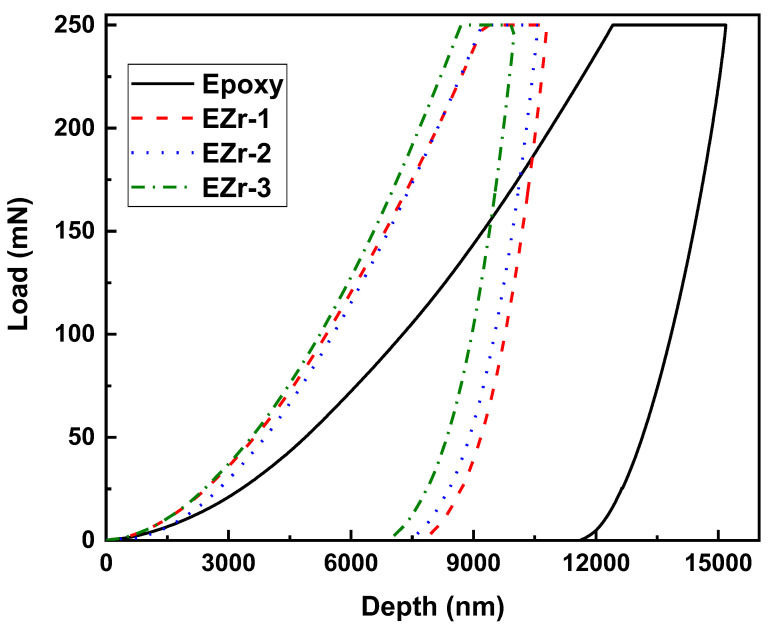
Load vs. depth curves for unfilled and ZrO_2_-filled epoxy coatings.

Nanoindentation was used to further analyze how adding ZrO_2_ nanoparticles affected the produced coatings’ mechanical properties. Indentations were carried out on all the prepared coatings utilizing a load management program. The coatings underwent strain against the Berkovich-type indenter up to a 250 mN load (maximum). The result of the nanoindentation test was the creation of a graph (Figure 4) showing the relationship between the applied force and the corresponding penetration depth during two different stages, loading and unloading. During the nanoindentation test, the penetration depth increased as a function of the applied load. In the nanoindentation curve, during the loading process, both types of deformation (elastic and plastic) occur; therefore, the loading portion is representative of both deformations, while after reaching maximum load, the unloading cure represents only elastic behavior. This unloading curve is used to calculate the elastic modulus.

With polymeric materials, a short-term creep was necessary to increase the reliability of hardness and elastic modulus results acquired from nanoindentation. When reaching the desired maximum load, the load is held for a certain period of time, which causes a further increase in depth at this holding stage. This increase in depth is because of the epoxy’s viscoelastic nature. This creep behavior influences the maximum depth and slope of the upper portion of the unloading load. As a result, if this creep is not considered, It will affect the final calculated results [19].

All the coatings were tested using similar loading conditions. The obtained load vs. depth curves depict a smooth loading process without any discontinuities or significant jumps in depth values until the maximum load for all the coatings is reached. The addition of NPs was responsible for shifting the depth to lower values, indicating an increase in properties as the coatings are capable of holding more load without allowing the indenter to penetrate deep into the coatings [20]. Upon reaching the highest defined load (250 mN), the maximum depth recorded for neat epoxy was 12,413 nm. The addition of ZrO_2_ in the percentages of 1, 2, and 3% resulted in lower depth values, which were recorded to be 9249, 9216, and 8701 nm, respectively.

The analysis for the obtained load vs. depth curves was performed with the machine-provided software, which analyze the hardness and reduced modulus value using the Oliver–Pharr model [21]. The following equations shown below give the details about how the hardness and elastic modulus were extracted.

(1) $$H=\frac{F_{max}}{A}$$

(2) $$E_{r}=\frac{1-\upsilon^{2}}{E}+\frac{1-\upsilon_{i}^{2}}{Ei}$$

where H represents hardness, F_max_ is the maximum load applied, A is the projected contact area at maximum load, E is sample elastic modulus, Poisson’s ratio (υ = 0.35 for polymer samples), E_r_ is the reduced modulus (obtained from indentation data), Ei is the indenter modulus (1141 GPa), and Poisson’s ratio of the diamond indenter (υ_i_ = 0.07) [22,23].

**Figure 5:**
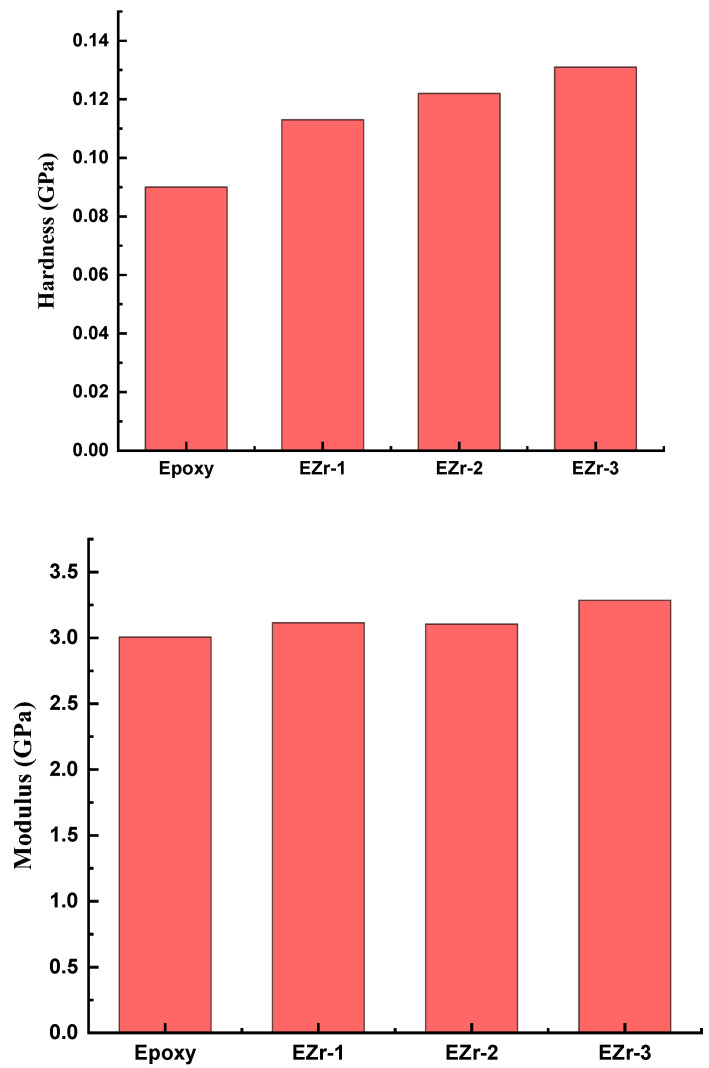
Graphical representation of obtained hardness and elastic modulus for all the coatings.

Figure 5 shows the obtained hardness and elastic modulus for all the coating samples. It can be seen from Figure 5 that the addition of ZrO_2_ nanoparticles has a positive influence on the hardness of the coating. With the increasing percentage, the hardness of the coating is improved. For the unfilled epoxy, the hardness value obtained was 0.09 GPa; with the addition of a maximum of 3 wt.% nanoparticles, it reaches 0.131 GPa. This is approximately a 45% increase in hardness, while there was no significant improvement in the elastic modulus of the coatings, where unfilled epoxy has an elastic modulus of 3.00 GPa and 3.28 GPa was recorded for coating with 3 wt.% addition, an increase of only 9%.

The increase in nanoparticle percentage increases hardness due to the reduction in free volume and higher cross-linked density. The decrease in free volume restricts the chain movement upon indentation. Because nanoparticles make it difficult to be affected by outside forces, their inclusion in epoxy causes an improvement in coating qualities. Because of its smaller size, there is a possibility of the indenter landing in an area where particles were agglomerated, resulting in higher hardness and lower elastic modulus properties [24,25].

### 3.4. EIS Data

**Figure 6:**
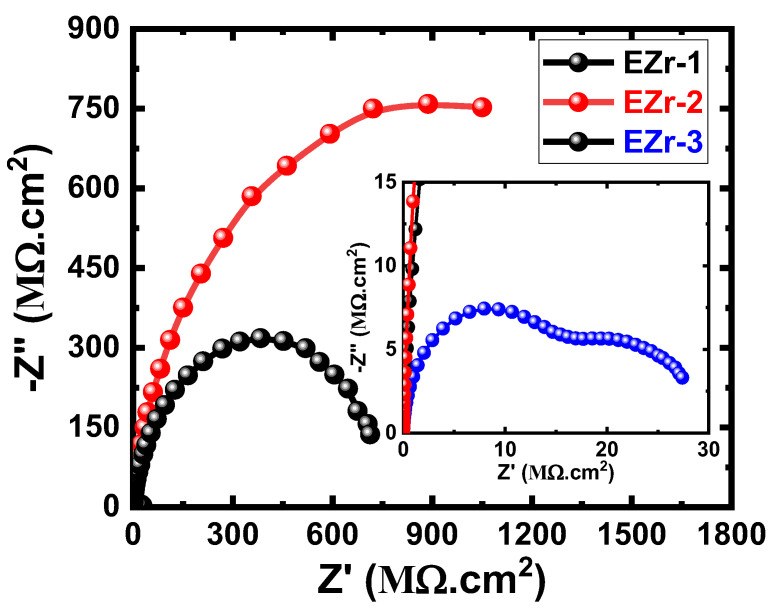
Nyquist plots for EZr samples for 1 d in the chloride solution.

**Figure 7:**
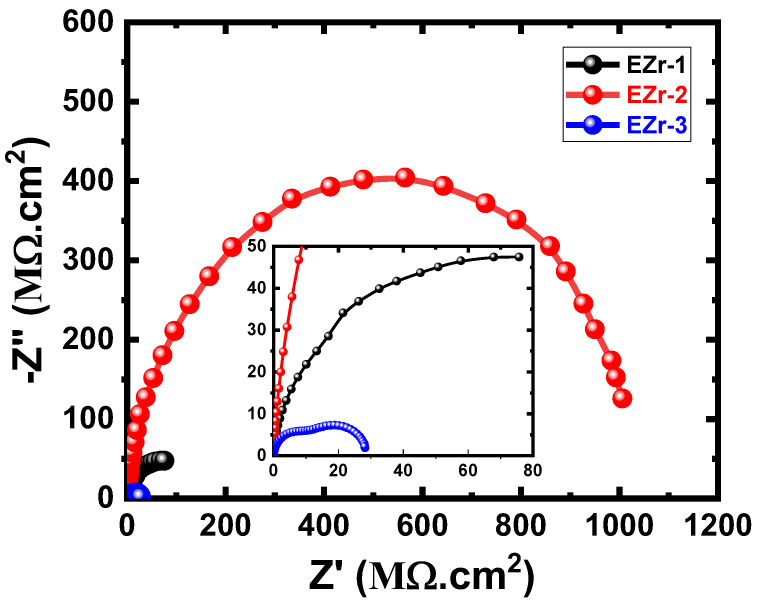
Nyquist plots for EZr samples for 7 days in the chloride solution.

**Figure 8:**
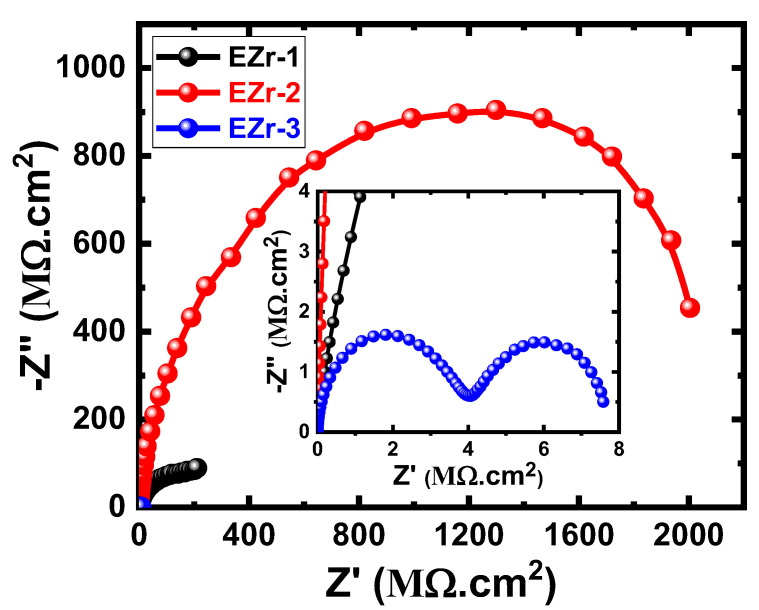
Nyquist plots for EZr samples for 14 days in the chloride solution.

**Figure 9:**
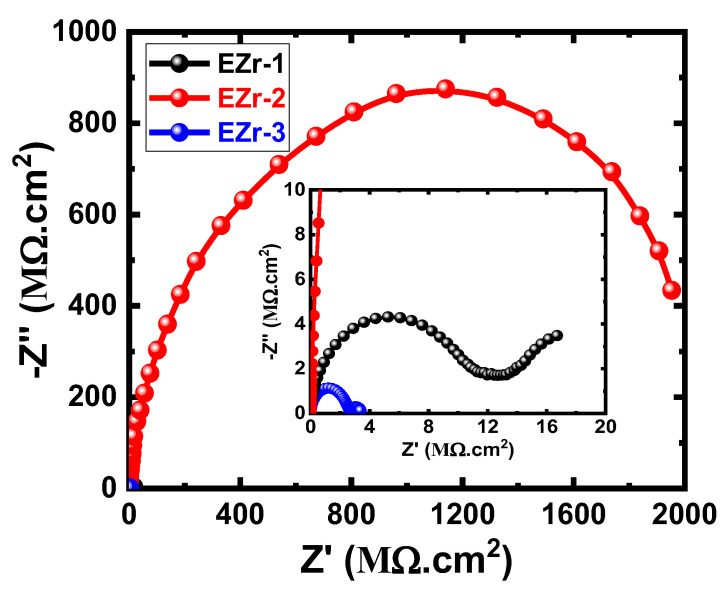
Nyquist plots for EZr samples for 21 days in the chloride solution.

**Figure 10:**
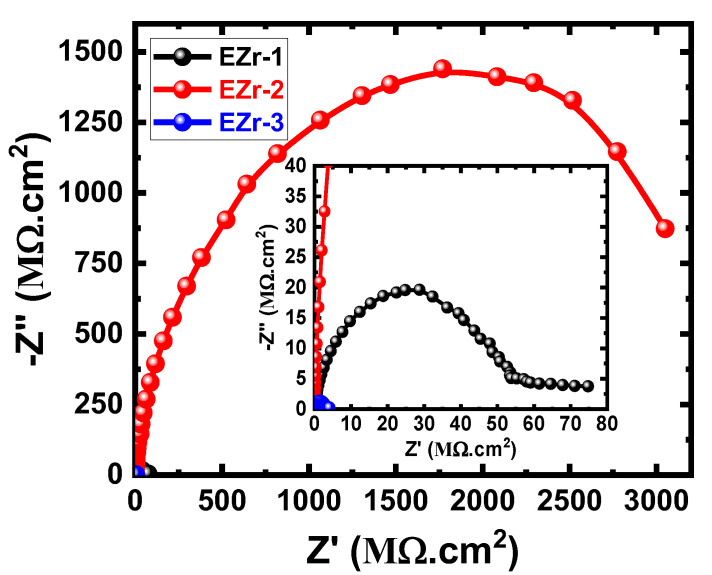
Nyquist plots for EZrs samples for 30 days in the chloride solution.

The EIS method has been reported as a powerful technique in studying the prevention of corrosion using coatings [23,26,27,28,29,30,31,32]. We have employed EIS to report the performance of the fabricated coatings against corrosion after various exposure times in NaCl electrolytes. Figure 6 shows the Nyquist plots for the coated steel samples (EZr-1, EZr-2, and EZr-3) after their exposure in 3.5% NaCl for 1 d before measurement. Similar plots were also obtained after 7, 14, 21, and 30 d, as shown in Figure 7, Figure 8, Figure 9 and Figure 10.

**Figure 11:**
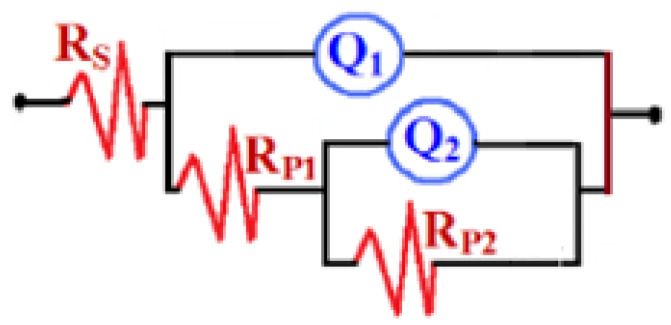
The employed circuit model.

Our EIS data have been fitted to the circuit model that is seen in Figure 11, and the values obtained from this circuit are listed in Table 5. Here, the symbols of the circuit of Figure 11 can be defined according to the universal definition as follows: R_S_ is the solution resistance, Q_1_ is the first constant phase elements (CPEs), R_P1_ is the first polarization resistance at the interface of the steel panels and the coatings, Q_2_ is a second constant phase element, and R_P2_ is another polarization resistance at the interface of the coating layers and NaCl solution.

The spectrum of the EZr-1 Nyquist plot depicted in Figure 6 shows only one small, distorted semicircle. Increasing Zr in the coating to 2% showed the widest diameter for the obtained semicircles. On the other hand, the Nyquist plot obtained with further increasing the percentage of Zr to 3% shows two semicircles; the first one is wide, while the other is a bit smaller. The diameter of the two semicircles obtained from EZr-3 coating is still the smallest. This indicates that increasing the percentage of Zr in the coating to 3% (in the EZr-3-coated steel panel) may lead to the formation of different layers on the surface of the coating during its immersion in NaCl solution for 1 d. The EIS data under these conditions thus clearly reveal that 2% Zr increased the corrosion resistance of the coating to its maximum, and this effect decreased when Zr increased to 3%.

The plots obtained after 7 days, as seen in Figure 7, showed that all coated panels have the same behavior as the ones obtained after 1 d (Figure 6). It is clear that the data obtained after 7 days (Figure 7) have smaller diameters compared to the diameters shown in Figure 6 for EZr-1 and EZr-2 samples. Only the EZr-3 sample showed almost the same behavior with almost the same diameter of the semicircle as the one obtained after 24 h. The decrease in the diameter of the semicircle is due to the decrease in the corrosion resistance, which, in turn, results from the degradation of the coating and the formation of new phases due to the long immersion period of time in the test solution. Further increasing the content of Zr to 2% under this condition, the obtained plot shows the highest and widest diameter for the obtained semicircle. The highest Zr% used here (3%) showed the lowest size of the obtained semicircles. This confirms the results obtained after 24 h that the best Zr content for higher corrosion resistance is 2% Zr, while using a lower (EZr-1) or higher (EZr-3) content decreases the corrosion resistance of the coating.

Further prolonging the exposure period to 14 days before measurement affects the impedance behavior for all samples. The Nyquist plots depicted in Figure 8 show that the spectra obtained for EZr-1 and EZr-3 coatings have much lower resistances, while the EZr-2 coating has higher resistance when compared to their behavior after 1 day and 7 days. The degradation of the EZr-1 and EZr-3 coatings due to the long immersion time is the reason for the decrease in the resistances. Here, EZr-2 coating does not produce the same degradability because of its higher corrosion resistance, which might have come from the concentration of zirconia and ZrO_2_ within the EZr-2 coating, making them the optimal nanoparticles that present the maximum coating stability.

After 21 days, as shown in Figure 9, the presence of 1% Zr led to the appearance of a semicircle and a segment. This behavior was not shown for shorter immersion periods such as 1 d and 7 d and is due to the formation and thickening of the corrosion products leading to the increase in corrosion resistance of the surface of coatings. Increasing the percentage of Zr to 2% highly increases the diameter of the corresponding Nyquist plot. On the other hand, the coating of 3% Zr showed the smallest diameter and confirms that increasing Zr% higher than 2% decreases the resistance to corrosion for the fabricated coatings. This was further confirmed by increasing the exposure time to 30 d before measurement, where the highest corrosion resistance was for the EZr-2 sample, followed by EZr-1, and the lowest was EZr-3. It is also proved that prolonging the time of immersion increases the corrosion resistance for all coatings, as seen from the increased range of values for both “Z’” and “–Z” seen from the Nyquist plots shown in Figure 6, Figure 7, Figure 8, Figure 9 and Figure 10. According to the listed values in Table 5, the highest values of R_S_, R_P1_, and R_P2_ were obtained for the EZr-2 formulae. On the other hand, the values of Y_Q1_ and Y_Q2_ (with their *n* values above 0.5 and close to unity) may be considered as double-layer capacitors. All results obtained from EIS plots and data recorded in Table 5 indicate that the corrosion resistance for the fabricated epoxy coatings increases in the following order: EZr-2 > EZr-1 > EZr-3. In addition, the exposure period, when increased from 1 d up to 30 d, increases the resistance to corrosion for the coatings in the same order.

## 4. Conclusions

The epoxy coatings incorporated with zirconia (ZrO_2_) nanoparticles indicated that the surfaces of particles with high positive charges supported corrosion stability. The deposition of zirconia particles and their incorporation in the epoxy coating was successfully performed, as confirmed by the energy-dispersive X-ray fluorescence spectroscopy. The amount of zirconium was also estimated in the epoxy coating. The acquired FT-IR spectra for the coatings indicate the chemical interaction of ZrO_2_ nanoparticles with oxygen and nitrogen atoms in the epoxy chains. The TGA experiments for coatings recorded the lowest weight loss values. The results indicated that the epoxy coating containing ZrO_2_ nanoparticles has better adhesion and slower degradation due to its low porosities. Among all the fabricated ZrO_2_ nanoparticle coatings, the coating with 2% Zr (EZr-2) presented an excellent corrosion resistance, depicting the highest impedance values compared to the other samples, EZr-1 and EZr-3.

## Figures and Tables

**Table 1:** Formulating ingredients of coatings prepared with ZrO_2_ NPs.

Sample	* Epoxy (gm)	MIBK (mL)	Xylene (mL)	ZrO_2_ (wt%)	Silane (gm)	* D-450 (gm)
EZr-1	83.34	10	10	1	2	16.66
EZr-2	83.34	10	10	2	2	16.66
EZr-3	83.34	10	10	3	2	16.66

* Epoxy resin and hardener (D-450) are under a stoichiometric balance.

**Table 2:** The obtained EDS results for coating-prepared coating samples.

Element/Coating	EZr-1	EZr-2	EZr-3
Carbon (C)	69.64%	69.40%	69.25%
Oxygen (O)	29.47%	29.15%	27.78%
Zirconium (Zr)	0.89%	1.45%	2.97%
Total	100	100	100

**Table 3:** Thermal stability data for the nanoparticles incorporated epoxy coatings.

Sample	15% Loss Temp. (°C)	25% Loss Temp. (°C)	50% Loss Temp. (°C)	75% Loss Temp. (°C)	Residue (%)
EZr-1	278.88	370.43	414.71	436.24	10.01
EZr-2	326.93	374.48	418.33	442.47	11.98
EZr-3	323.34	374.76	418.24	440.84	12.89

**Table 4:** Mechanical properties obtained with different percentages of nanoparticles.

Sample	DFT (µm)	Pendulum Hardness *	Scratch (Kg)	Impact (lb/in^2^)
EZr-1	100 ± 10	149	7.0	80
EZr-2	100 ± 10	145	7.5	88
EZr-3	100 ± 10	149	8.5	80

* Oscillations calculated on coating’s surface.

**Table 5:** EIS parameters obtained for the different coatings in the sodium chloride solutions.

Samples	R_S_/Ω cm^2^	Q_1_		R_P1_/MΩcm^2^	Q_2_		R_P2_/MΩcm^2^
		Y_Q1_/MΩ cm^2^	n_1_		Y_Q2_/MΩ cm^2^	n_2_	
EZr1 (1d)	33.7	267	0.981	135	353	0.765	321
EZr2 (1d)	42.0	369	0.959	227	185	0.751	865
EZr3 (1d)	30.6	333	0.975	196	348	0.685	293
EZr1 (7d)	45.2	336	0.974	169	338	0.675	355
EZr2 (7d)	47.8	545	0.976	228	296	0.607	910
EZr3 (7d)	42.0	421	0.971	202	330	0.699	327
EZr1 (14d)	37.8	394	0.860	197	310	0.638	403
EZr2 (14d)	49.5	601	0.981	256	278	0.634	2400
EZr3 (14d)	43.5	432	0.961	217	324	0.626	351
EZr1 (21d)	41.2	479	0.995	239	322	0.609	384
EZr2 (21d)	51.5	813	0.980	299	263	0.649	2240
EZr3 (21d)	43.3	619	0.972	234	316	0.602	426
EZr1 (30d)	42.0	511	0.895	241	299	0.655	420
EZr2 (30d)	56.0	785	0.869	273	238	0.689	4010
EZr3 (30d)	45.8	608	0.982	255	292	0.620	443

## Data Availability

The data presented in this study are available on request from the corresponding author.
